# Re-Evaluating Platelet-Rich Plasma Dosing Strategies in Sports Medicine: The Role of the “10 Billion Platelet Dose” in Optimizing Therapeutic Outcomes—A Narrative Review

**DOI:** 10.3390/jcm14082714

**Published:** 2025-04-15

**Authors:** Alessandro Corsini, Loris Perticarini, Stefano Palermi, Pierfrancesco Bettinsoli, Andrea Marchini

**Affiliations:** 1 Genoa Cricket and Football Club, 16155 Genova, Italy; 2 Fondazione Poliambulanza Istituti Ospedalieri, 25125 Brescia, Italy; loris.perticarini@gmail.com; 3 Department of Medicine and Surgery, UniCamillus-Saint Camillus International University of Health Sciences, 00187 Rome, Italy; stefano.palermi@unicamillus.org; 4 Istituto Clinico Sant’Anna, 25125 Brescia, Italy; pierfrancesco.bettinsoli@gmail.com; 5 Juventus FC, 10151 Torino, Italy; andrea.marchini@jmedical.eu

**Keywords:** platelet-rich plasma, platelet dose, sport medicine, orthobiologic

## Abstract

Platelet-rich plasma (PRP) therapy is increasingly recognized as a promising treatment for musculoskeletal disorders, including osteoarthritis (OA), tendinopathy, and muscle injuries. This narrative review synthesizes the current literature to evaluate the efficacy of PRP, with a focus on platelet dosing strategies, leukocyte composition, and preparation protocols. Evidence suggests that optimal therapeutic outcomes are achieved when platelet doses exceed 3.5 billion per injection, with cumulative doses of 10–12 billion across multiple treatments. In intra-articular applications, leukocyte-poor PRP (LP-PRP), characterized by reduced neutrophil content, demonstrates superior efficacy compared to leukocyte-rich PRP (LR-PRP). However, its effectiveness in tendon and muscle regeneration remains a subject of debate. Preliminary data suggest that the inclusion of peripheral blood mononuclear cells (PBMNCs) may enhance PRP efficacy, though robust clinical trials are required to confirm these findings. Furthermore, red blood cell contamination and pre-activation have been identified as detrimental to PRP effectiveness, highlighting the need for standardized preparation protocols. This review emphasizes the importance of tailoring PRP formulations to patient-specific factors and musculoskeletal conditions. Future research should focus on refining PRP preparation techniques, identifying optimal leukocyte compositions, and establishing standardized guidelines to enhance clinical outcomes.

## 1. Introduction

Platelet-rich plasma (PRP) therapy has gained increasing attention as promising regenerative treatment for musculoskeletal disorders, including osteoarthritis (OA), tendinopathy, and muscle injuries [1]. As an autologous biologic therapy, PRP is widely used in sports medicine to promote tissue regeneration, reduce inflammation, and accelerate recovery [1,2]. Despite its growing adoption, significant variability in preparation methods, platelet dosing strategies, and leukocyte composition has led to inconsistent clinical outcomes [3]. The lack of standardized protocols and well-defined efficacy parameters remains a significant challenge in optimizing PRP applications [2,3].

PRP has been utilized for over 30 years in musculoskeletal medicine, yet scientific evidence supporting its efficacy is often inconsistent due to methodological limitations in clinical studies. A systematic review of 124 clinical trials found that only 12.1% provided comprehensive protocol descriptions, and 26.6% quantitatively reported PRP composition [4]. Moreover, an analysis of nearly 500 PRP-related publications revealed that 44% contained methodological errors, with 26% deemed unreliable [5]. These findings highlight the need for PRP preparation and application standardization to ensure reproducibility and therapeutic efficacy.

One of the most critical determinants of PRP effectiveness is platelet dose, with emerging evidence suggesting that higher platelet dose correlates with better clinical outcomes [6,7]. Studies indicate that intra-articular PRP treatments exceeding 3.5 billion platelets per injection and cumulative doses of 10–12 billion platelets across multiple sessions yield the most significant improvements in pain relief and functional recovery [3,6,7]. Additionally, leukocyte composition plays a pivotal role in determining PRP’s therapeutic potential [2,3]. While leukocyte-poor PRP (LP-PRP) demonstrates superior efficacy in intra-articular applications due to its reduced inflammatory profile [8,9,10,11,12], its role in tendon and muscle repair remains debated [8,13,14]. Recent investigations suggest that including peripheral blood mononuclear cells (PBMNCs) may enhance PRP’s regenerative properties, but further clinical validation is necessary [15,16,17,18,19,20,21,22,23].

This narrative review aims to synthesize current evidence on PRP therapy for musculoskeletal injuries, emphasizing key factors influencing treatment outcomes, including platelet dose, leukocyte composition, and preparation protocols. By examining recent literature, this review seeks to provide a clearer understanding of how to optimize PRP formulations and guide future research toward establishing standardized treatment protocols.

## 2. Materials and Methods

This narrative review synthesizes existing literature on PRP therapy for musculoskeletal disorders, specifically OA, tendinopathy, and muscle injuries. It highlights current knowledge, key trends, and considerations for optimizing PRP preparation and application. The review adheres to established methodological guidelines for narrative reviews, ensuring a comprehensive and structured approach to data selection and analysis.

### 2.1. Literature Search Strategy

A structured search was conducted using the following electronic databases: PubMed, Scopus, and Web of Science. The search strategy included combinations of medical subject headings (MeSHs) and free-text terms related to PRP therapy, given as follows:

“Platelet-rich plasma”, “PRP”, “osteoarthritis”, “tendinopathy”, “muscle injuries”, “leukocyte-rich PRP”, “leukocyte-poor PRP”, “platelet dose”, and “red blood cell contamination”.

A manual review of reference lists from relevant publications was also performed to identify additional studies not captured in the database search. Only articles published between 2000 and 2024 were included to ensure a comprehensive evaluation of PRP therapy, allowing for both foundational and recent findings to be considered.

### 2.2. Inclusion and Exclusion Criteria

Studies were selected based on their relevance to PRP therapy in musculoskeletal disorders, specifically on preparation methods, platelet dosing, leukocyte content, and therapeutic outcomes.

#### 2.2.1. Inclusion Criteria

- Peer-reviewed articles published in English.
- Studies discussing PRP composition, preparation, and clinical application in OA or tendinopathy.
- Reports providing quantitative or qualitative data on platelet concentration, leukocyte content, or PRP efficacy.

#### 2.2.2. Exclusion Criteria

- Case reports, opinion pieces, or non-peer-reviewed literature.
- Studies focusing on acute injuries or conditions unrelated to OA or tendinopathy.
- Papers that lack detailed descriptions of PRP preparation, platelet composition, or treatment outcomes.

### 2.3. Analysis Approach

All selected articles were critically evaluated based on the following:

- Study design, methodology, and inclusion criteria.
- PRP preparation protocols, including platelet dosing, leukocyte composition (LP-PRP vs. leukocyte-rich PRP—LR-PRP), red blood cell contamination, and activation methods.
- Reported outcomes, including pain relief, functional improvements, and biological effects on musculoskeletal tissues.

Identified trends, discrepancies, and methodological inconsistencies were systematically analyzed and contextualized within the broader body of literature. The review aims to provide evidence-based recommendations for optimizing PRP therapy in sports medicine and musculoskeletal disorders.

## 3. The Platelet Dose in PRP Therapy: A Key Determinant of Efficacy

### 3.1. Variability in PRP Systems and the Need for Standardization

Several factors, including platelet dose, concentration, biological composition, and preparation quality, determine PRP therapy’s effectiveness in musculoskeletal disorders. To achieve clinically significant and lasting benefits, PRP must contain an optimal number of platelets, capable of releasing bioactive factors that drive tissue repair and regeneration [3,6,7]. However, increasing interest in PRP has led to over 50 different PRP preparation systems, each producing PRP with varying platelet concentrations, cellular compositions, and formulations [24,25,26,27,28,29,30,31,32,33,34,35,36,37,38]. This variability contributes to inconsistencies in patient outcomes, as reflected in numerous studies [2,3,7,28,32,39,40].

While higher platelet concentrations are often associated with greater bioactive factor release, research suggests that total platelet dose—rather than platelet concentration alone—is a more reliable predictor of therapeutic success [6,7,37,41,42,43]. Other factors influence clinical outcomes, such as PRP injection volume, initial blood draw volume, and leukocytes or red blood cells. Consequently, a standardized approach to PRP preparation and dosing is necessary to improve the reproducibility of results and ensure optimal treatment efficacy.

### 3.2. The Impact of Platelet Dose on PRP Efficacy

#### 3.2.1. Defining the Minimum Effective Platelet Dose

The first clinical study to establish a dose–response relationship in PRP therapy was conducted by Everts et al. [3], who demonstrated that a minimum effective platelet dose of >3.5–4 × 10^9^ platelets per injection is necessary for clinical benefits across various soft tissue conditions. Similarly, Berger et al. [44] found that higher platelet concentrations enhanced tenocyte proliferation and migration in a dose-dependent manner, further supporting the notion that platelet dose—not just concentration—is crucial for therapeutic success. Further evidence from Bansal et al. [41] demonstrated that treating knee OA patients with a PRP injection delivering a total platelet dose of 10 billion resulted in sustained improvements in pain relief, function, and inflammatory marker reduction over 12 months. Additionally, a randomized controlled trial (RCT) by Patel et al. [42] compared two doses of LP-PRP in early-stage knee OA (Kellgren–Lawrence (KL) classification: stage 1: doubtful narrowing of joint space, possible osteophyte formation; stage 2: definite osteophytes, possible joint space narrowing KL2):

- 2.8 billion platelets (4 mL PRP) vs. 5.6 billion platelets (8 mL PRP).
- Both groups showed clinical improvement, but patients receiving the higher platelet dose exhibited superior pain and function outcomes over six months.

These findings suggest that higher platelet doses are associated with more significant clinical benefits, particularly in degenerative joint diseases such as knee OA. However, PRP is not recommended for hip OA due to inconsistent evidence.

#### 3.2.2. Validating the 4 Billion Platelet Threshold

Recent studies have sought to determine the minimum effective platelet dose required to achieve clinical success. De Matthaeis et al. [37] investigated 212 patients with KL1-3 knee OA treated with 4 billion platelets per injection in a neutrophil-depleted, PBMNC-enriched PRP formulation. The study reported the following:

- Outcome Measures in Rheumatology-Osteoarthritis Research Society International (OMERACT-OARSI) responder rates of 68.9% at 3 months, 72.7% at 6 months, and 70.6% at 12 months.
- Significant improvements in Visual Analogic Scale (VAS) and Western Ontario and McMaster Universities Arthritis Index (WOMAC) scores.
- The KL2 group showed the best results in pain reduction and WOMAC scores at 6 months.
- The study concluded that high-dose neutrophil-depleted PRP is effective for KL1-3 (KL 3: multiple osteophytes, definite joint space narrowing, mild bone sclerosis) knee OA, potentially relieving symptoms for up to a year and slowing disease progression.

Similarly, the RESTORE RCT [45] compared a total dose of 1.6 × 10^9^ platelets (325 × 10^3^/μL, 5 mL PRP) to a saline control in patients with KL2-3 knee OA. The study found no significant clinical difference between PRP and saline at 12 months, confirming that PRP doses below 4 billion platelets are ineffective.

A systematic review by Berrigan et al. [6] analyzed 29 RCTs on PRP for knee OA, examining 31 PRP treatment arms:

- 28 PRP treatment arms (90%) with doses >5.5 billion platelets reported significant clinical improvements.
- Studies with ≤2.3 billion platelets failed to demonstrate therapeutic benefits (*p* < 0.01).
- The review identified an ideal cumulative dose of 10 billion platelets across multiple injections for optimal efficacy.

These findings reinforce the importance of platelet doses in PRP therapy, suggesting clinicians should aim for a minimum dose of 4 billion platelets per injection and a cumulative dose of 10 billion platelets across multiple treatments.

### 3.3. PRP Efficacy Across Different Musculoskeletal Conditions

While most research on platelet dose has focused on OA, several studies have examined PRP’s effectiveness in tendinopathies, muscle injuries, and other musculoskeletal disorders. Meta-analyses and RCTs have reported that higher platelet doses improve treatment efficacy across various conditions:

- Rotator cuff tendinopathy: PRP doses > 6 billion platelets led to better pain relief and functional recovery [46]
- Lateral epicondylitis: Studies with 5–10 billion platelets consistently demonstrated significant pain reduction [47,48]
- Gluteal tendinopathy: 5–10 billion platelets were associated with better patient-reported outcomes [49,50]
- Patellar tendinopathy: PRP doses <5 billion platelets resulted in no significant difference from placebo, suggesting a placebo effect or insufficient dosing [51,52]
- Achilles tendinopathy and plantar fasciitis: Both conditions showed positive clinical outcomes, even with lower platelet doses (<5 billion platelets) [53,54]
- Muscle injuries: Conflicting evidence exists; no conclusive data support PRP’s ability to reduce reinjury risk or accelerate return to play [55].

Interestingly, Raum et al. [56] proposed that platelet-poor plasma may have unique regenerative properties for muscle injuries, potentially inducing [4,5,57] myoblast differentiation and enhancing native muscle regeneration. However, further clinical validation is needed.

### 3.4. Discussion: The Future of Platelet Dose Standardization in PRP Therapy

Given the strong correlation between platelet dose and clinical outcomes, there is a clear need to standardize PRP preparation protocols. Studies consistently show that platelet doses below 4 billion per injection are mainly ineffective, while doses exceeding 10 billion across multiple treatments produce the most consistent and sustained improvements. However, challenges remain:

- Variability in PRP preparation devices: Differences in platelet yield, leukocyte composition, and red blood cell contamination significantly impact PRP quality and efficacy [24,25,26,27,28,29,30,31,32,33,34,35,36,37,38]
- Lack of standardized dosing guidelines: Many clinical studies fail to report precise PRP compositions, making and comparing findings and optimizing difficult treatments [4,5,57]
- Need for patient-specific approaches: The optimal platelet dose may vary depending on the treated condition, the patient’s age, and the severity of the pathology [7].

To address these challenges, future research should focus on developing evidence-based dosing guidelines, ensuring that PRP formulations are tailored to maximize therapeutic potential. Additionally, clinicians should report platelet doses in clinical studies, improving trial comparability and reproducibility.

## 4. The Platelet Dose and Analgesic Effect

### 4.1. Platelet Dose and Its Role in Pain Modulation

Recent evidence suggests that platelets directly correlate with PRP’s analgesic effect [1,12]. The mechanism by which PRP alleviates pain remains complex, involving the modulation of inflammatory mediators, tissue regeneration, and neuroplastic changes in pain pathways.

The first clinical observation of PRP’s analgesic properties was reported by Everts et al. [58] in 2008, who noted that PRP significantly reduced post-surgical pain in patients undergoing subacromial decompression. Since then, numerous studies have shown that PRP treatments can lead to substantial pain relief or even complete pain elimination in patients with musculoskeletal injuries [59,60]. However, not all studies have confirmed these benefits, with some reporting minimal or no pain relief [61,62]. These discrepancies highlight the critical role of platelet dose, PRP formulation, and administration techniques in determining the consistency of PRP’s analgesic effects [41,63].

Beyond platelet dose, other factors influencing PRP’s pain modulation include the following:

- Injury type and tissue composition (e.g., cartilage vs. tendon vs. muscle).
- Route of administration (intra-articular vs. intramuscular vs. perineural).
- PRP activation method (exogenous activation vs. endogenous platelet activation).
- Leukocyte concentration (LP-PRP vs. LR-PRP).

### 4.2. Clinical Evidence Supporting PRP’s Analgesic Effects

Several studies have demonstrated PRP’s long-term pain relief potential in different musculoskeletal conditions:

- Kuffler et al. [64] studied PRP for neuropathic pain in patients with damaged and unrepaired nerves. They observed that PRP-treated patients experienced progressive pain relief over three weeks, with some patients reporting pain elimination for over six years.
- A systematic review by Johal et al. [40] concluded that PRP consistently reduces pain across multiple orthopedic indications, reinforcing its role in pain management.
- Yoshida et al. [65] explored PRP’s analgesic properties in a rat model, demonstrating that a platelet concentration of 1.0 × 10^6^/μL resulted in complete pain relief. In contrast, a 50% lower concentration produced significantly less pain reduction.

These findings suggest that higher platelet doses may amplify PRP’s analgesic properties, but further research is needed to determine the optimal dose for pain management.

### 4.3. LP-PRP vs. LR-PRP: Which Provides Better Pain Relief?

Emerging data suggest that LP-PRP may provide superior analgesic effects compared to LR-PRP. A meta-analysis of 24 RCTs involving 1344 patients concluded that LP-PRP offers significantly greater pain relief than LR-PRP in knee OA [12]. This is consistent with studies demonstrating that leukocytes in PRP can contribute to inflammation, potentially counteracting PRP’s analgesic effects [8].

## 5. To Be or Not to Be Rich: Leukocyte-Poor Platelet-Rich Plasma (LP-PRP) vs. Leukocyte-Rich Platelet-Rich Plasma (LR-PRP)

### 5.1. The Debate over Leukocyte Enrichment in PRP

The impact of leukocyte concentration in PRP therapy remains a topic of debate. Depending on the PRP preparation system used, leukocyte levels can vary significantly, with some formulations being LR-PRP and others LP-PRP [24,25,26,27,28,29,30,31,32,33,34,35,36,37,38,66,67].

Several studies indicate that LP-PRP is superior to LR-PRP for intra-articular treatments, particularly OA [8,9,10,11].

- Riboh et al. [11] conducted a meta-analysis of six RCTs and three prospective studies (1055 patients). They found that LP-PRP resulted in significantly better WOMAC scores than hyaluronic acid and placebo, whereas LR-PRP did not improve substantially.
- A meta-analysis of 24 RCTs [12] confirmed that LP-PRP provides greater pain relief than LR-PRP in knee OA.
- A recent double-blind RCT [68] involving 132 patients found no difference between LP-PRP and LR-PRP in clinical outcomes, though LR-PRP provided no additional benefits.

These findings suggest that reducing neutrophil content in PRP formulations may enhance therapeutic efficacy, particularly in cartilage-related conditions.

### 5.2. Neutrophils vs. Monocytes: Understanding Leukocyte Function in PRP

One of the key challenges in the LP-PRP vs. LR-PRP debate is that not all leukocytes function similarly.

- Neutrophils are highly inflammatory and may release cytokines and matrix metalloproteinases that promote pro-inflammatory and catabolic effects, potentially detrimental to tissue healing [69,70].

Monocytes/macrophages, on the other hand, exhibit pro-reparative properties and play a key role in immune modulation and tissue regeneration [15,16,17,18,19,20,21,22,71,72,73,74,75,76].

This distinction has led researchers to explore monocyte-enriched PRP formulations that minimize neutrophils while enhancing the regenerative M2 macrophage phenotype [37].

### 5.3. PBMNC-Enriched PRP: A Novel Approach to Enhance PRP Efficacy

Recent research suggests that PBMNCs—which include monocytes, macrophages, and lymphocytes—may enhance PRP’s regenerative properties.

- PBMNC-enriched PRP has shown promising results in cartilage regeneration [17,18,19,20,71,72,73,77,78] and tendon healing [21,22,75,76,79].
- PBMNCs also play a crucial role in muscle repair [74,80,81,82,83,84].
- Macrophage plasticity is essential for tissue healing, as M1 macrophages (inflammatory) can transition to M2 macrophages (anti-inflammatory and reparative) [21,22,85,86,87,88].

### 5.4. Clinical Evidence for PBMNC-Enriched PRP in OA Treatment

Two recent clinical trials have evaluated PBMNC-enriched PRP in osteoarthritis:

- De Matthaeis et al. [37] conducted a retrospective clinical trial in KL1-3 knee OA using a high-dose, neutrophil-poor, PBMNC-enriched PRP formulation (12 billion platelets over three injections, two weeks apart). The study reported the following:
  OMERACT-OARSI responder rates of 68.9% (3 months), 72.7% (6 months), and 70.6% (12 months).
  Significant improvements in VAS and WOMAC scores compared to baseline.

- Saita et al. [89] confirmed similar results using PBMNC-enriched PRP for knee OA.

These findings suggest that PRP formulations enriched with monocytes and PBMNCs may offer superior regenerative potential compared to standard LP-PRP or LR-PRP, as previously observed on anterior cruciate ligament [90].

### 5.5. Discussion: Optimizing PRP Formulations for Clinical Success

While the optimal PRP formulation for different conditions remains under investigation, we can draw the following suggestions from the current evidence:

- LP-PRP is preferable for intra-articular treatments, especially OA, due to its anti-inflammatory properties.
- Monocyte-enriched PRP may enhance tissue repair by promoting M2 macrophage-driven regeneration.
- Neutrophil-rich PRP (LR-PRP) should be used cautiously, as excessive inflammation may hinder healing under certain conditions.

Future research should focus on the following:

- Identifying the ideal balance between leukocyte subtypes (monocytes vs. neutrophils) in PRP formulations.
- Standardizing PRP preparation protocols to improve reproducibility across clinical studies.
- Exploring PBMNC-enriched PRP as a potential next-generation orthobiologic therapy.

## 6. Red vs. Yellow PRP: The Impact of Red Blood Cell Contamination

### 6.1. Why Red Blood Cells Should Be Minimized in PRP

While the debate over leukocyte concentration in PRP formulations remains unresolved, there is a consensus that RBC contamination should be minimized. Unlike platelets and leukocytes, RBCs do not contribute to tissue regeneration and may be detrimental to musculoskeletal tissues [91,92].

The toxic effects of RBCs in PRP occur due to the following:

- Hemolysis: RBCs break down, releasing hemoglobin, hemin, and iron, which can trigger oxidative stress, inflammation, and immune suppression [91].
- Cytotoxicity: Joint Hemosiderin accumulation has been linked to cartilage damage [93].
- Eryptosis: A process similar to apoptosis in RBCs, eryptosis leads to the release of macrophage migration inhibitory factor, which inhibits monocyte/macrophage recruitment, stem cell migration, and fibroblast proliferation [91].
- Microvascular dysfunction: RBC degradation products lead to vasoconstriction and impaired circulation, which may hinder tissue healing [91].

Thus, PRP preparation protocols should aim to minimize RBC contamination. This is especially important in intra-articular applications, where RBC-free PRP (Yellow PRP) is preferred over RBC-contaminated PRP (Red PRP) [92].

### 6.2. PRP Preparation Techniques and RBC Contamination

The RBC content in PRP depends on the centrifugation method, collection layer, and device used. Different PRP preparation systems yield varying degrees of RBC contamination, which influences the biological activity of the final PRP product [24,25,26,27,28,29,30,31,32,33,34,35,36,37,38].

PRP devices can be broadly categorized into two groups:

1. Leukocyte-poor, RBC-free PRP systems separate and discard RBCs while preserving a high platelet yield. However, they often produce lower platelet concentrations (<400,000 platelets/μL) and total doses (<2 billion platelets). Some require a second centrifugation (double-spin method) to increase platelet concentration.
2. Leukocyte-rich PRP systems: These devices target the buffy coat to collect higher platelet counts. However, they may also include higher RBC and neutrophil contamination, which can introduce pro-inflammatory effects [4,5,24,66,94].

Additionally, blood draw volume plays a critical role in PRP composition.

- Low blood volume draws (10–20 mL) often result in lower platelet recovery.
- Larger blood draws (50–60 mL) generally allow for higher platelet doses, but the final composition depends on the centrifugation efficiency and collection technique [24,28,31,32].

Given this variability, standardized RBC removal techniques are necessary to ensure optimal PRP formulations for clinical use.

## 7. Essential Traits and Minimal Clinical Platelet Dose of Point of Care PRP Device

### 7.1. Why Platelet Dose Must Be Reported in PRP Studies

The clinical effectiveness of PRP therapy is significantly influenced by platelet dose, yet many studies report only platelet concentration or enrichment factors rather than absolute platelet numbers [4,5,14,24,26].

- Platelet yield (recovery rate): The percentage of platelets collected from whole blood into the final PRP product.
- Platelet dose (total platelets delivered per injection): A more reliable predictor of PRP efficacy than concentration alone [3,6,7].

A study evaluating 20 different PRP preparation devices found that platelet doses ranged from 0.21 to 5.43 × 10^9^, depending on the system used [28]:

- None of the devices achieved 90% platelet recovery; some collected more RBCs than platelets.
- Devices with higher platelet yields often had higher RBC and leukocyte contamination.

### 7.2. The Impact of PRP Device Selection on Platelet Dose and RBC Contamination

PRP devices differ in the following aspects:

- Platelet recovery rates: Devices with low recovery rates (<60%) may result in subtherapeutic platelet doses [24,25,26,27,28,29,30,31,32,33,34,35,36,37,38].
- RBC contamination: Some devices collect high RBC concentrations, which can reduce PRP efficacy and safety [92,95].
- Leukocyte content variability: The final PRP preparation can have widely different leukocyte compositions, affecting inflammatory and regenerative properties [24,25,26,27,28,29,30,31,32,33,34,35,36,37,38].

Table 1 compares the most commonly used point-of-care PRP devices, classified according to DEPA rules [28].

## 8. Activated vs. Non-Activated PRP: Influence on Growth Factor Release

### 8.1. Mechanisms of PRP Activation

PRP contains platelets coated with glycoprotein receptors and adhesion molecules, which must be activated to release growth factors. PRP activation can occur through the following mechanisms:

- Exogenous activation: Addition of calcium chloride, calcium gluconate, or autologous thrombin.
- Endogenous activation: Contact with human type I collagen or exposure to tissue microenvironments.

Growth factor (GF) release must be sustained over time to maximize PRP’s regenerative effects.

- Rapid activation (exogenous methods) may cause early GF depletion, reducing PRP’s long-term therapeutic benefits [96,97,98,99]
- Slow, tissue-dependent activation (endogenous methods) promotes gradual GF release, supporting longer-lasting biological activity [96,100].

### 8.2. Clinical Studies on Activated vs. Non-Activated PRP

Non-activated PRP enhances stem cell proliferation: Studies have shown that non-activated, pH-buffered PRP increases mesenchymal stem cell (MSC) proliferation five-fold [100]. Pre-activated PRP may reduce chondrogenesis and osteogenesis: In vitro studies indicate that pre-activated PRP decreases cartilage and bone formation, whereas non-activated PRP enhances tissue regeneration [99].

## 9. The Influence of Gender and Age on PRP Composition

### 9.1. Gender Differences in PRP Composition

PRP composition can vary significantly between male and female patients, affecting the growth factor levels and cytokine release [101]

- Male PRP: Higher levels of Platelet-derived growth factor-BB (PDGF-BB), Vascular endothelial growth factor (VEGF), and Transforming growth factor-beta 1 (TGF-β1), along with pro-inflammatory cytokines (IL-1β, IRAP, TNF-α).
- Female PRP: Lower levels of pro-inflammatory cytokines, but no significant difference in overall platelet concentration.

### 9.2. Age-Related Differences in PRP Efficacy

Elderly patients tend to have lower insulin-like growth factor 1 (IGF-1) levels, which may impact PRP’s anabolic effects on tissues [101]. Younger patients may have more excellent MSC responsiveness, potentially enhancing PRP’s regenerative properties.

### 9.3. Gender Differences in PRP Clinical Outcomes

A study of 418 patients (529 knees) receiving three weekly PRP injections found the following findings [102]:

- Women had a higher success rate (59.6%) than men (50.9%).
- Despite experiencing more severe symptoms initially, women showed more significant symptom improvement, leading to similar long-term outcomes between genders.

These findings highlight the importance of personalizing PRP therapy based on patient demographics and biological variability.

## 10. Discussion

PRP therapy has gained widespread use in musculoskeletal medicine, particularly OA, tendinopathies and muscle injuries. However, significant variability in PRP formulations, platelet dose, leukocyte composition, and preparation methods have led to inconsistent clinical outcomes. This narrative review highlights the key determinants of PRP efficacy and provides recommendations based on current evidence (Table 2).

### 10.1. Platelet Dose and PRP Efficacy

One of the most important determinants of PRP efficacy is platelet dose. While platelet concentration has traditionally been used as a marker of PRP quality, growing evidence suggests that total platelet dose per injection is a more reliable predictor of therapeutic success [3,6,7,37,41,42,43]. Everts et al. [3] first suggested a minimum effective dose of 3.5–4 × 10^9^ platelets, while Bansal et al. [41] and Patel et al. [42] demonstrated that higher platelet doses (>5 billion) yield superior pain relief and functional improvements. Conversely, the RESTORE RCT [45] found that low-dose PRP (1.6 billion platelets) was ineffective, reinforcing the need for adequate dosing.

### 10.2. PRP and Pain Modulation

PRP’s analgesic effects are linked to growth factor release and inflammatory modulation, with higher platelet doses providing stronger pain relief [1,12,27]. Studies by Kuffler et al. [64] and Yoshida et al. [65] indicate that higher platelet counts contribute to long-term pain reduction. At the same time, meta-analyses suggest that LP-PRP outperforms LR-PRP in pain relief [12].

### 10.3. Leukocyte Content: LP-PRP vs. LR-PRP

The debate over LR-PRP vs. LP-PRP remains unresolved, though LP-PRP has been shown to be more effective for intra-articular injections due to its lower inflammatory response and greater chondrogenic potential [8,9,10,11]. While neutrophils may drive excessive inflammation, monocytes/macrophages in PBMNC-enriched PRP appear to enhance tissue repair [1,15,37,65,89,90,103]. De Matthaeis et al. [37] and Saita et al. [89] showed that PBMNC-enriched PRP improves outcomes in knee OA, suggesting a shift toward monocyte-based formulations.

### 10.4. Red Blood Cell (RBC) Contamination in PRP

Unlike leukocytes, RBCs are universally detrimental, promoting oxidative stress, tissue damage, and immune suppression [91,92,93]. RBC contamination leads to hemolysis, oxidative stress, and cytotoxic effects, particularly in intra-articular injections. The presence of RBCs can also impair monocyte/macrophage recruitment and stem cell migration, reducing PRP’s regenerative potential. Standardizing PRP preparation techniques to eliminate or significantly reduce RBC contamination is essential, particularly for OA and cartilage regeneration applications.

### 10.5. PRP Activation: Exogenous vs. Endogenous Activation

The method of PRP activation significantly impacts growth factor release kinetics and biological activity. While exogenous activation (using calcium chloride, thrombin, etc.) induces rapid platelet degranulation, it may lead to the premature depletion of bioactive factors [96,100]. In contrast, endogenous activation (via contact with tissue collagen) promotes a more sustained release of growth factors, potentially enhancing long-term therapeutic effects [96].

### 10.6. Gender- and Age-Related Variability in PRP Composition and Response

Emerging evidence suggests that PRP composition varies based on gender and age [101,102]. Male PRP tends to have higher levels of inflammatory cytokines, while female PRP may have a more balanced regenerative profile. Additionally, older patients may exhibit reduced PRP efficacy due to lower IGF-1 levels and diminished stem cell responsiveness.

Personalizing PRP formulations based on patient demographics could help optimize clinical outcomes.

### 10.7. Future Directions

To enhance PRP efficacy, future research should seek to achieve following:

- Standardize PRP dosing protocols to improve clinical consistency.
- Investigate monocyte-enriched PRP for its regenerative potential.
- Optimize PRP activation strategies for sustained growth factor release.
- Address demographic variations in PRP composition and response.

By refining these aspects, PRP therapy can become a more reliable and effective regenerative treatment in sports medicine and orthopedics.

## 11. Conclusions

PRP therapy holds great potential for treating musculoskeletal conditions, but standardization is crucial to optimizing outcomes. This review highlights that platelet dose is a critical determinant of efficacy, with 4 billion platelets per injection being the minimum effective threshold and 10 billion across multiple treatments yielding the best results. Additionally, LP-PRP appears superior to LR-PRP for intra-articular applications, while PBMNC-enriched PRP may offer new regenerative advantages. Future research should focus on defining optimal PRP formulations, eliminating RBC contamination, and personalizing treatments based on patient-specific factors. By addressing these issues, PRP therapy can become a more effective and standardized regenerative treatment in sports medicine and orthopedics.

## Figures and Tables

**Table 1:** Comparative table of PRP systems with PRP type [24,25,26,27,28,29,30,31,32,33,34,35,36,37,38].

PRP System	Blood Volume (mL)	ACD (mL)	PRP Volume (mL)	Plts Enrichment Factor (Average)	Platelet Recovery Efficiency (%) Mean Value	Platelet Concentration (×10^6^/µL) Mean Value	Platelet Dose (Billions Plts) Mean Value	Centrifugation	RBC Contamination	PRP Type	Neutrophils	PBMNC	Refs.
Magellan	25–60	4–6	3–6	5.5 x	65	780	5.4	Double spin	yes	LR-PRP	yes	yes	[24,25,27,28,30]
Mini GPS III	27	3	3.0	4 X	49.2	1015	2.8	Double spin	yes	LR-PRP	yes	yes	[29,31,33]
GPS II	55	5	6	2.3 X	22.9	1076	3.6	Double spin	yes	LR-PRP	yes	yes	[26,27,28,29,30]
Smartprep Terumo	60	6	7–10	4 X	65	720	4.9	Double spin	yes	LR-PRP	yes	yes	[28,30]
Hy Tissue 20 Fidia	18	2	Single spin 4–8 mL Double spin 2–3 mL	2–3X	83.5 75–81	288–497 863–968	2-2.5 2.4-2.9	Single spin Double spin	no no	LP-PRP	no no	no no	[34,35,36], internal data
Arthrex ACP	13	2	3 2–5 4 4.8	1.3 X	29.5	357–390 357 372	1.25	Single spin	no	LP-PRP	no	no	[12,24,26,28,29,30,31,32,33]
Tropocells 22 Estar Medical	20	2 (buffered pH 7.4)	4.0	5 X	85	1080	4	Single spin	no	LP-PRP	no	Yes	[37,38]
RegenLab BCT	8	1	3	0.95 X 0.59 X	82	142.5	1.35	Single spin	no	LP-PRP	no	no	[20,21,28,29,30,32,33]
Endoret BTI	9	1	2	1.3	48	150	1	Single spin	no	LP-PRP	no	no	[28,30]

Protocol, volume collected, and volume obtained from each preparation system provided in publications [24,25,26,27,28,29,30,31,32,33,34,35,36,37,38]. PRP volume, Plts enrichment factors, recovery, concentration, and dose are the mean value.

**Table 2:** Key recommendations for the use of PRP.

Parameter	Recommendation	LoE
Platelet dose in OA	Aim for 5–10 billion platelets per injection for optimal efficacy	1A
Minimum platelet dose threshold	PRP doses below 4 billion platelets per injection are ineffective	1A
Cumulative platelet dose	Total dose of 10+ billion platelets across multiple injections improves outcomes	2A
LP-PRP vs. LR-PRP for OA	LP-PRP is preferred for intra-articular injections due to lower inflammation	1A
Leukocyte concentration in PRP	Neutrophil-rich PRP may prolong inflammation, while monocyte-enriched PRP may enhance healing	2A
PBMNC-enriched PRP	PBMNC-enriched PRP may provide superior tissue regeneration	2B
RBC contamination	PRP should be as RBC-free as possible to avoid cytotoxic effects	1A
PRP activation method	Endogenous activation (via tissue contact) promotes sustained growth factor release	2A
PRP for pain modulation	Higher platelet doses (>4 billion) enhance PRP’s analgesic effects	2A
Gender differences in PRP	Male PRP has higher pro-inflammatory cytokines, while female PRP has a more balanced regenerative profile	2B
Age-related PRP variability	Older patients may require PRP formulations with enhanced IGF-1 support	2B
Standardization of PRP devices	Clinicians should report platelet dose rather than just platelet concentration	1A
Optimizing PRP for different conditions	PRP formulations should be personalized based on tissue type and patient demographic	2B

LOE: level of evidence.

## Data Availability

Original data are available on reasonable request to the corresponding author.
